# Development of stable S-scheme 2D–2D g-C_3_N_4_/CdS nanoheterojunction arrays for enhanced visible light photomineralisation of nitrophenol priority water pollutants

**DOI:** 10.1038/s41598-024-52950-3

**Published:** 2024-02-05

**Authors:** Muhammad Saad, Ali Bahadur, Shahid Iqbal, Sajid Mahmood, Muhammad Tayyab, Matar Alshalwi, Mazloom Shah

**Affiliations:** 1 Centre for Organic and Nanohybrid Electronics, Silesian University of Technology, Konarskiego 22B, 44-100 Gliwice, Poland; 2 Joint Doctoral School, Silesian University of Technology, Akademicka 2A, 44-100 Gliwice, Poland; 3 Department of Chemistry, School of Natural Sciences (SNS), National University of Science and Technology (NUST), H-12, Islamabad, 46000 Pakistan; 4 Department of Chemistry, College of Science, Mathematics, and Technology, Wenzhou-Kean University, Wenzhou, 325060 Zhejiang Province China; 5 Dorothy and George Hennings College of Science, Mathematics and Technology, Kean University, 1000 Morris Ave, Union, NJ 07083 USA; 6 Nottingham Ningbo China Beacons of Excellence Research and Innovation Institute, University of Nottingham Ningbo China, Ningbo, 315100 China; 7 Functional Materials Group, Gulf University for Science and Technology, 32093 Mishref, Kuwait; 8 Department of Chemical and Life Sciences, Qurtuba University of Science and Information Technology, Dera Ismail Khan, Pakistan; 9 Department of Chemistry, College of Science, King Saud University, PO Box 2455, Riyadh, 11541 Saudi Arabia; 10 Department of Chemistry, Faculty of Science, Grand Asian University Sialkot, Punjab, Pakistan

**Keywords:** Catalysis, Environmental chemistry

## Abstract

The investigation focused on creating and studying a new 2D–2D S-scheme CdS/g-C_3_N_4_ heterojunction photocatalyst. Various techniques examined its structure, composition, and optical properties. This included XRD, XPS, EDS, SEM, TEM, HRTEM, DRS, and PL. The heterojunction showed a reduced charge recombination rate and more excellent stability, helping to lessen photocorrosion. This was due to photogenerated holes moving more quickly out of the CdS valence band. The interface between g-C_3_N_4_ and CdS favored a synergistic charge transfer. A suitable flat band potential measurement supported enhanced reactive oxygen species (ROS) generation in degrading 4-nitrophenol and 2-nitrophenol. This resulted in remarkable degradation efficiency of up to 99% and mineralization of up to 79%. The findings highlighted the practical design of the new 2D–2D S-scheme CdS/g-C_3_N_4_ heterojunction photocatalyst and its potential application in various energy and environmental settings, such as pollutant removal, hydrogen production, and CO_2_ conversion.

## Introduction

Nitrophenols are a persistent group of pollutants commonly found in wastewater, originating from various industrial activities^1^. The National Pollutant Release Inventory (NPRI) of Canada^2^ has identified nitrophenols as priority contaminants and have established surface water quality standards limiting their concentration to 1 part per billion (ppb)^3^. Long-term exposure to nitrophenols causes respiratory issues, muscle weakness, tremors, skin/eye/mucous membrane irritation, and damage to various animal organ systems, including fetal development^4^. Thus, drinking water's maximum allowable nitrophenol concentration is less than 1 ppm^5^. Due to their chemical stability, nitrophenols are not easily removed from water and tend to form toxic byproducts. Complete mineralization, i.e., these pollutants' breakdown into their constituent elements, is therefore necessary and requires green energy sources^5^. To achieve this, finding efficient and effective photocatalysts for the degradation of nitrophenols is critical in environmental remediation. The utilization of low-cost semiconductor materials, water, and sunlight to generate reactive oxygen species (ROS) for effectiveness and economic feasibility is an ideal solution^6,7^.

Moreover, strict maintenance of operating conditions can be challenging and may lead to the formation of more toxic intermediates^8–10^. However, higher charge recombination, photocorrosion, and the broadband gap of most catalysts restrict their full use as much of the sunlight spectra is in the visible area^11,12^. Therefore, developing and optimizing stable photocatalysts that have high ROS production efficiency and are capable of utilizing the visible spectrum for nitrophenol mineralization is crucial. Photocatalysis is a potent technique for environmental remediation, particularly for water purification. CdS has been extensively studied as a photocatalyst owing to its ability to absorb photons, attributable to its fine band gap of 2.41 eV^13^. However, its photocatalytic capability is reduced due to decreased active sites and increased charge recombination, leading to instability and corrosion of the catalyst under illumination^14^. Li et. al., specifically worked on the CdS based photocatalysts and produced its composites like Ta_3_N_5_/CdS core–shell S-scheme based nanofibers^15^, S-scheme hetero-structured Bi_2_MoO_6_/Cd_0.5_Zn_0.5_S microspheres/carbon dots^16^, and Cd_0.5_Zn_0.5_S nanoparticles onto Bi_2_WO_6_ microspheres^17^ for its enhanced photocatalytic applications. Zhongliao et al.^18^, reported Step-scheme CdS/TiO_2_ and used it for enhanced photocatalytic CO_2_ reduction. Bicheng et al^19^, reported 2D g-C_3_N_4_ based heterojunction for improved photocatalysis. Several earth-abundant photocatalytic systems driven by visible light have been developed to remove Nitrophenols partially or entirely from water. These systems include CdS/Carbon/MoS_x_^20^, Co_3_O_4_ loaded WO_3_^21^, N-Doped Reduced Graphene-CdS^22^, p-type Mn_3_O_4_/ZnO^23^, CeO_2_^24^, V_2_O_5_^25^, CuO/ZnO^26^, and V_2_O_5_/N,S–TiO_2_^27^. However, most reported work suffers from issues such as low degradation or partial removal, catalyst corrosion, and the use of either artificial light sources or large band gap materials with limited solar spectrum utilization. Additionally, N-Doped Reduced Graphene-CdS and CdS/Carbon/MoSx catalysts were reported without considering the percentage of achieved mineralization.

In many narrow band gap semiconductors, a swift recombination rate of e^−^–h^+^ pairs is generated by photoexcitation. However, CdS experiences photo-corrosion owing to the unstable S^2−^ species, which can be readily oxidized by photoinduced h^+^^28–30^. Literature has widely examined this phenomenon, and it is well established that the photo-corrosion of CdS results from the interaction between the semiconductor and the surrounding environment, such as the presence of oxygen or water molecules. There have been several research on this topic to investigate the mechanisms of photo-corrosion and to develop methods to mitigate its detrimental effects, including the use of protective coatings or the modification of the semiconductor surface chemistry^31–35^. These investigations have brought to light how crucial it is to thoroughly know and regulate the behavior of CdS-based devices in order to appreciate the intricate interaction between semiconductor characteristics and their surroundings.

The integration of an additional semiconductor material to create a heterojunction has been reported to be a viable approach to address the limitations inherent in CdS-based photocatalysts^35–37^. A fortuitous discovery reveals that the alignment of the valence and conduction bands of graphitic carbon nitride (g-C_3_N_4_) and cadmium sulfide (CdS) exhibits a highly compatible overlap of band energies, rendering them eminently suitable for the construction of heterostructures^38,39^. Further, the polymeric nature and structure of g-C_3_N_4_ will support and stabilize the composite material. Moreover, g-C_3_N_4_ establishes an electron transfer conduit that mitigates photogenerated electron–hole recombination, thereby amplifying the photoconversion efficiency of the CdS photocatalyst^40–42^. This translates to an increase in electron–hole pair separation efficiency, which augments photoactivity.

In this study, we look at the exceptional charge transfer partnership seen at the carefully tuned nano-joining point, which follows a specific S-Scheme electron transfer pathway as explained by Wu et al.^43^. Quanpeng et al.^44^, also reported the S-Scheme ZnS/TiO_2_ and used it for photocatalytic H_2_ production. Both CdS and g-C_3_N_4_ are advantageous because of their 2D shapes, which increase their surface areas and provide more structural stability. Also, the electron transport channel encourages the buildup of electrons within the cadmium sulfide conduction band and h^+^ in the g-C_3_N_4_ valence band, resulting in less S^2–^ oxidation-a leading cause of cadmium sulfide photo corrosion. This special electron transfer pathway creates electrons with more positive potential, helpful for making superoxide radicals and holes with increased negative potential that allow for the development of OH radicals, with the combined effect of these radicals leading to an increased photocatalytic breakdown ability of the g-C_3_N_4_/CdS catalyst. To the best of our knowledge, S-Scheme 2D–2D g-C3N4/CdS has not been reported for its applications in photocatalytic mineralization of nitrophenol based water pollutants. The catalysts tested in this study showed degradation successes of 84% and 99.4% and mineralization successes of 56.5% and 79% for 2-nitrophenol and 4-nitrophenol, respectively.

## Experimental section

### Synthesis

Cadmium acetate dihydrate (Cd. Ac), thiourea, ethylenediamine (EDA), melamine, and urea, all of the analytical grade, were acquired from suppliers and used without further treatment (see supplementary information S1. Reagents used). A typical synthesis included mixing 2 mmol of Cd. Ac, 6 mmol of thiourea, and 60 mL of EDA in an autoclave coated with Teflon and heating it to 100 °C for eight hours. After cooling, the mixture was centrifuged into separate components, cleaned with ethanol and water, and then let it dry for a whole night at 50 °C in a vacuum oven to produce soft, golden 2D CdS sheets. Urea and melamine were combined in a mortar and pestle to produce g-C_3_N_4_ in a single pot. The powder was then heated in a muffle furnace to 550 °C for two hours, with a 2.5 °C per minute temperature increase. Finally, g-C_3_N_4_/CdS composites with CdS concentrations of 5%, 10%, 15%, 20%, and 30% were prepared as shown in Figure S1.

### Characterisation

We obtained patterns by conducting XRD analyses using a Bruker D8 Advance diffractometer. We used a Thermo Fisher Scientific Tecnai G2 Spirit microscope, which was equipped with an EDAX Apollo X analyzer, to gather data for TEM, energy-dispersive X-ray (EDX) mapping and HRTEM. We employed a Shimadzu XPS-7000 multifunctional X-ray spectrometer for XPS measurements. Using a PerkinElmer Lambda 1050 spectrophotometer, we obtained the ultraviolet–visible (UV–vis) absorption spectra of the produced materials. We assessed steady-state photoluminescence (PL) spectra using an Edinburgh Instruments FLS1000 spectrofluorometer. We executed Mott-Schottky (MS) plot measurements on a Metrohm Autolab PGSTAT302N Electrochemical Workstation in a conventional three-electrode system at room temperature. The system used a platinum wire as the counter electrode, an Ag/AgCl as a reference electrode, and a photocatalyst-coated FTO as the working electrode. We examined nitrophenol samples both before and after photodegradation using a Shimadzu TOC-L TOC analyzer.

### Photocatalytic measurements

Batch investigations on fixed-bed photocatalytic degradation were carried out in Pyrex® glass reactors with dimensions of 125 × 30 mm in the open air. An optimized amount of 50 mg each of CdS, g-C_3_N_4_, g-C_3_N_4_/CdS in 100 mL of a 30 ppm phenolic substrate solution were separately stirred for 30 min to ensure optimal solubility, and to ensure the adsorption–desorption equilibrium in a dark environment. Subsequently, the suspension was exposed to sunlight (intensity: 900 ± 100 × 10^2^ lx) during fixed daytime intervals. Samples were collected at 10-min intervals to monitor the degradation and mineralization processes. Ultraviolet–visible (UV–Vis) spectroscopy and TOC analyses were utilized to assess the progress of these processes.

## Results and discussion

### *Physiochemical properties of g-C*_*3*_*N*_*4*_*/CdS nano-heterojunction*

**Figure 1:**
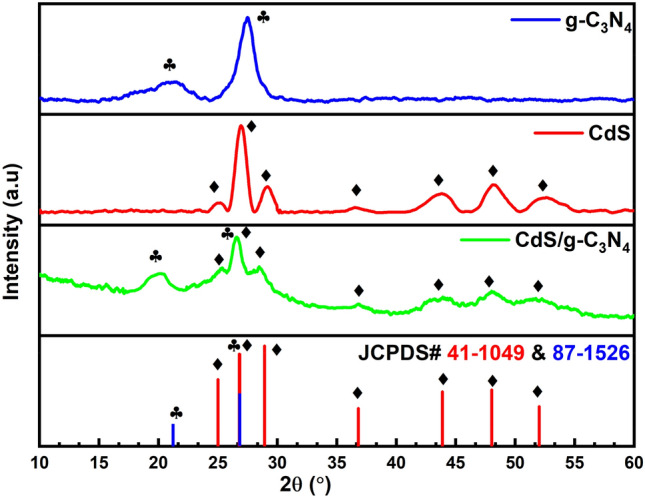
XRD Patterns of CdS, g-C_3_N_4_ and CdS/g-C_3_N_4_.

The insight into the crystallinity and phase parameters of g-C_3_N_4_, CdS, and CNCS samples was completed through XRD. The XRD patterns (Fig. 1) demonstrate that the six diffraction peaks of CdS hexagonal wurtzite structure (JCPDS No. 41-1049) may be allocated to the (100), (002), (101), (102), (110), (103), and (112) crystal planes at values of 24.8°, 26.5°, 28.2°, 36.6°, 43.7°, 47.8°, and 51.8° respectively. Compared with the JCPDS No. 87-1526, both the (100) planar structural packing motif of tristriazine units and the (002) graphite-like piling of g-C_3_N_4_ were the reason for the two diffraction peaks observed in pure g-C_3_N_4_ at 21.6° and 26.5°, respectively. The co-existence of all prominent peaks in the XRD of g-C_3_N_4_/CdS confirms the development of ultra-thin heterojunction between the pristine materials.

**Figure 2:**
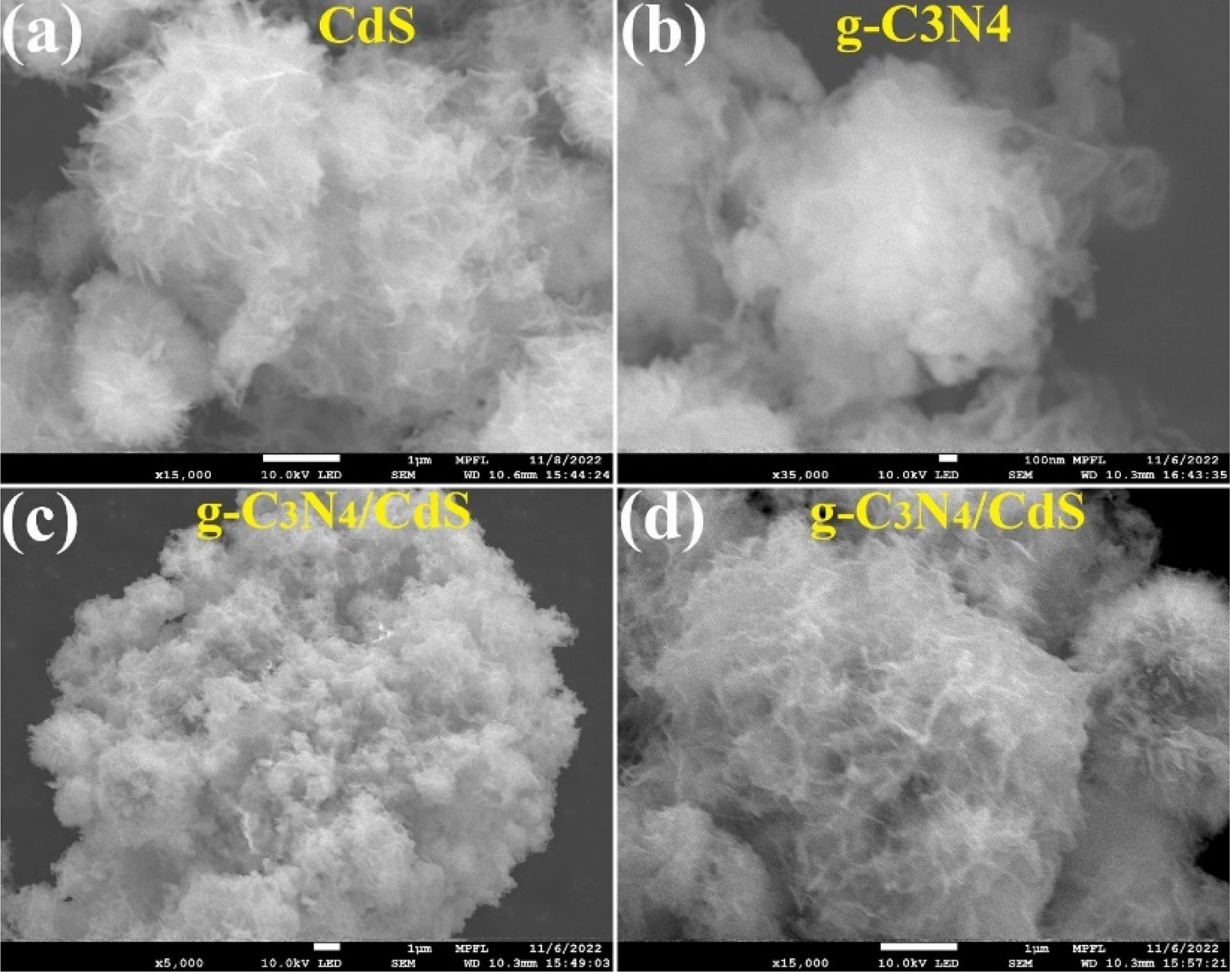
SEM profiles of (**a**) CdS (**b**) g-C_3_N_4_ (**c**,**d**) g-C_3_N_4_/CdS.

Scanning Electron Microscope (SEM) study provided information about the catalyst's outward shape and appearance. The figure shows CdS tending to form 2D flower-like structures (Fig. 2a) due to the highly flexible and thin sheets that make up its crystal lattice. The formation of such structures is related to the hexagonal wurtzite crystal structure of CdS, which is made up of thin, flexible sheets along certain crystallographic planes. On the other hand, pure g-C_3_N_4_ (Fig. 2b) comprised nanosheets with a laminar structure, giving it a silk veil-like appearance. The polymerization of melamine molecules results in a structure consisting of layers locked together by weak van der Waals forces, which is connected to this morphology (Fig. 2c,d).

**Figure 3:**
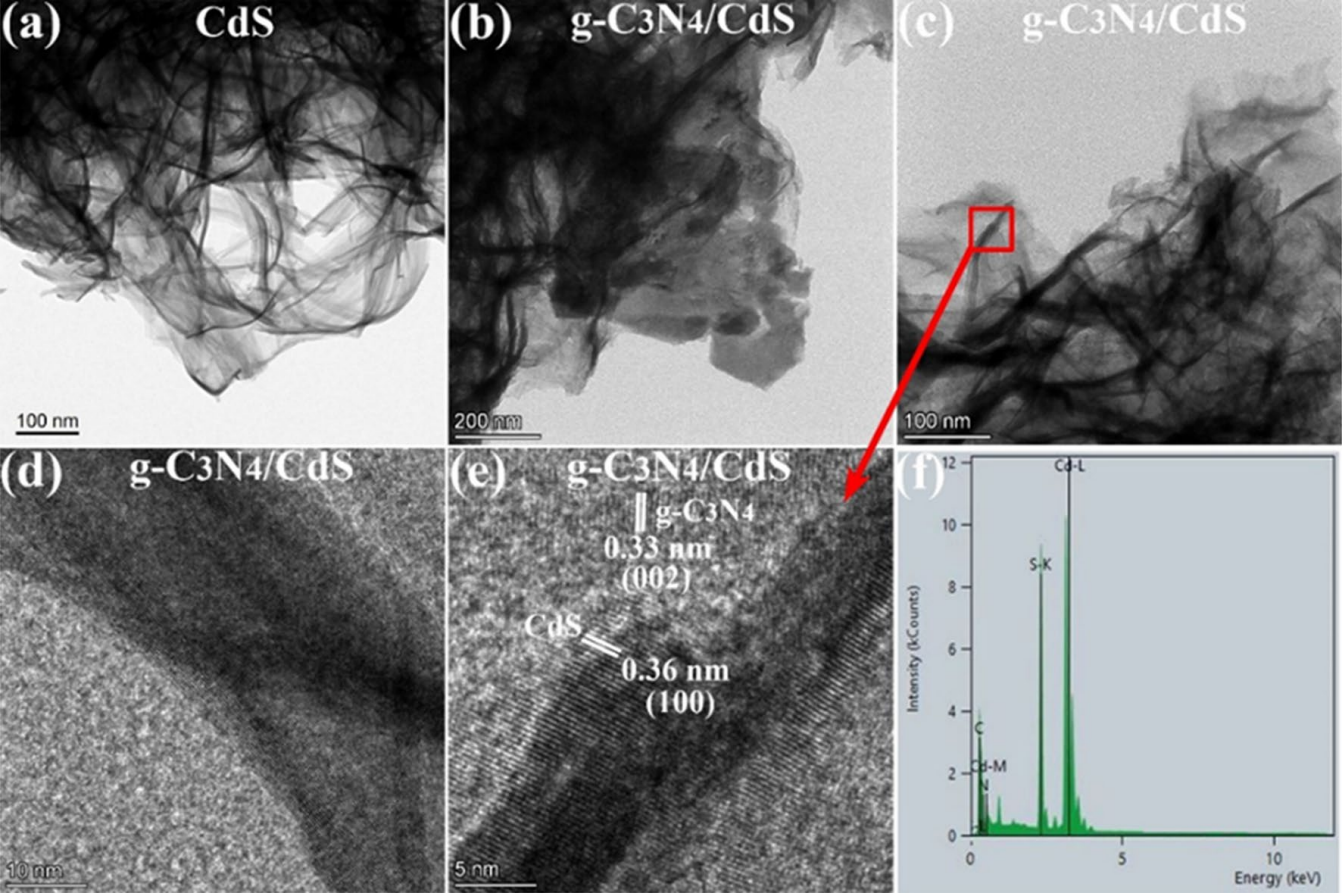
TEM images of (**a**) CdS (**b**,**c**) g-C_3_N_4_/CdS. HRTEM images (**d**, **e**) g-C_3_N_4_/CdS. EDS spectra (**f**) g-C_3_N_4_/CdS.

To further understand the internal composition and developmental mechanisms of the S-Scheme-g-C_3_N_4_/CdS heterojunction, this investigation used TEM, EDX spectroscopy and HRTEM. TEM images showed that the as-synthesized CdS (Fig. 3a) possesses almost transparent flexible 2D uniform ultrathin nanosheets (NSs) with a curly morphology sheet sheets' high flexibility and thinness are related to the hexagonal wurtzite crystal phase of CdS. The TEM (Fig. 3b,c) and HRTEM (Fig. 3d,e) images reveal that the CdS sheets are decorated with g-C_3_N_4_ sheets, forming nano-heterojunctions. The lattice spacing of 0.36 nm and 0.33 nm are allocated to the (100) and (002) crystal faces of CdS and g-C_3_N_4,_ correspondingly, indicating the formation of a crystallographic interface between the two materials. Due to the composites' greater specific surface area than pure CdS, the 2D CdS NSs provide anchor sites to immobilize the g-C_3_N_4_, which may partly inhibit its reaggregation. The EDS spectrum (Fig. 3f) shows peaks for Cd-L and Cd-M, indicating the presence of cadmium in the composite. The peak for S-K suggests the presence of sulfur, which is expected for CdS. Additionally, peaks for N and C confirm the existence of g-C_3_N_4_ in the catalyst. The existence of both materials creates a synergistic effect that can enhance their photocatalytic capability.

**Figure 4:**
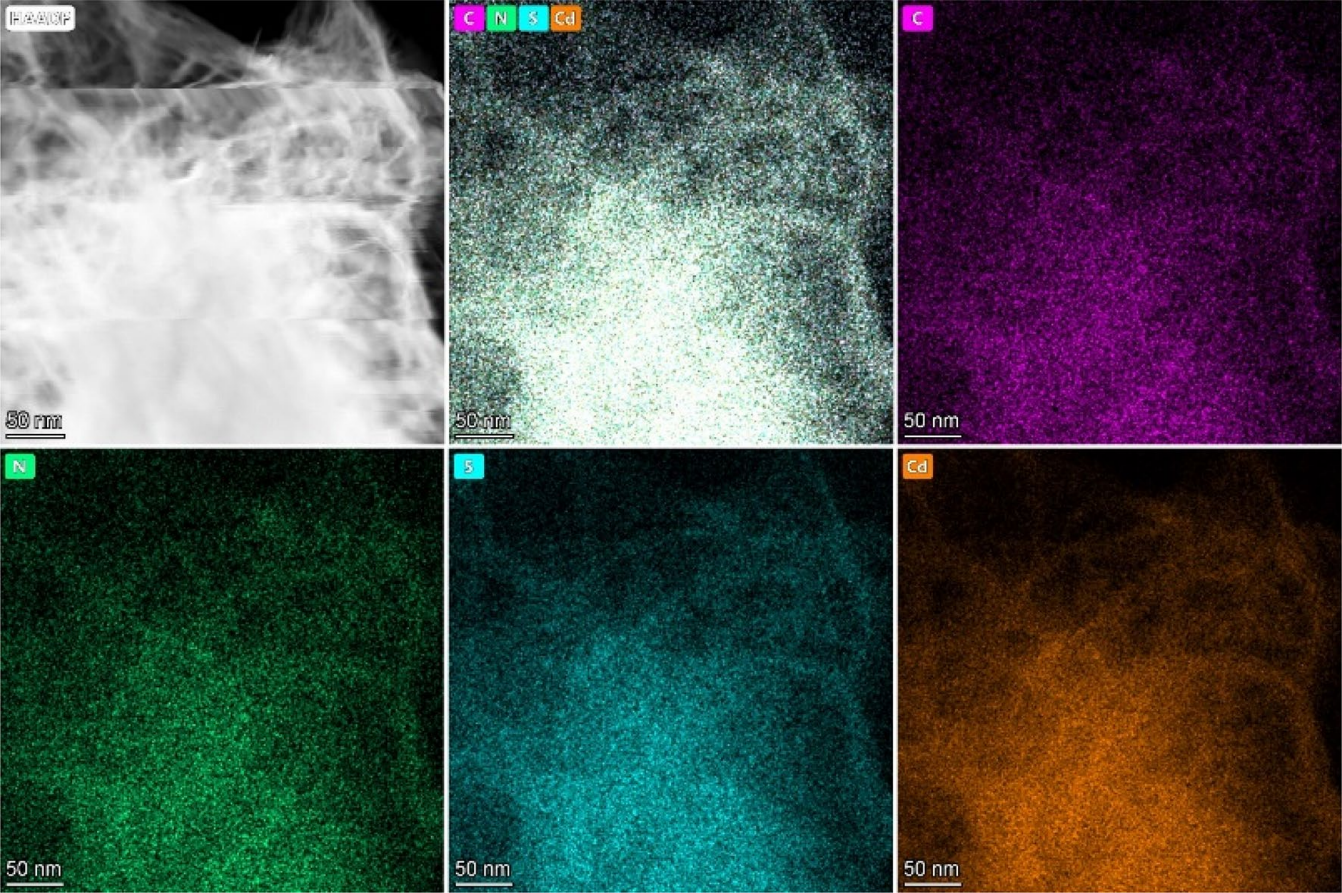
Elemental Mapping of g-C_3_N_4_/CdS.

The investigation of the elemental makeup and distribution of heterojunctions between g-C_3_N_4_ and CdS was conducted thoroughly using STEM-energy-dispersive X-ray spectroscopy (Fig. 4). For the g-C_3_N_4_/CdS composite, elemental mapping revealed an even distribution of cadmium (Cd), sulfur (S), carbon (C), and nitrogen (N) within the two-dimensional sheet-like structure. Such homogeneity implies adequate mixing and dispersion of g-C_3_N_4_ and CdS components, crucial for maximizing photocatalytic performance through enhanced energy transfer and charge separation.

**Figure 5:**
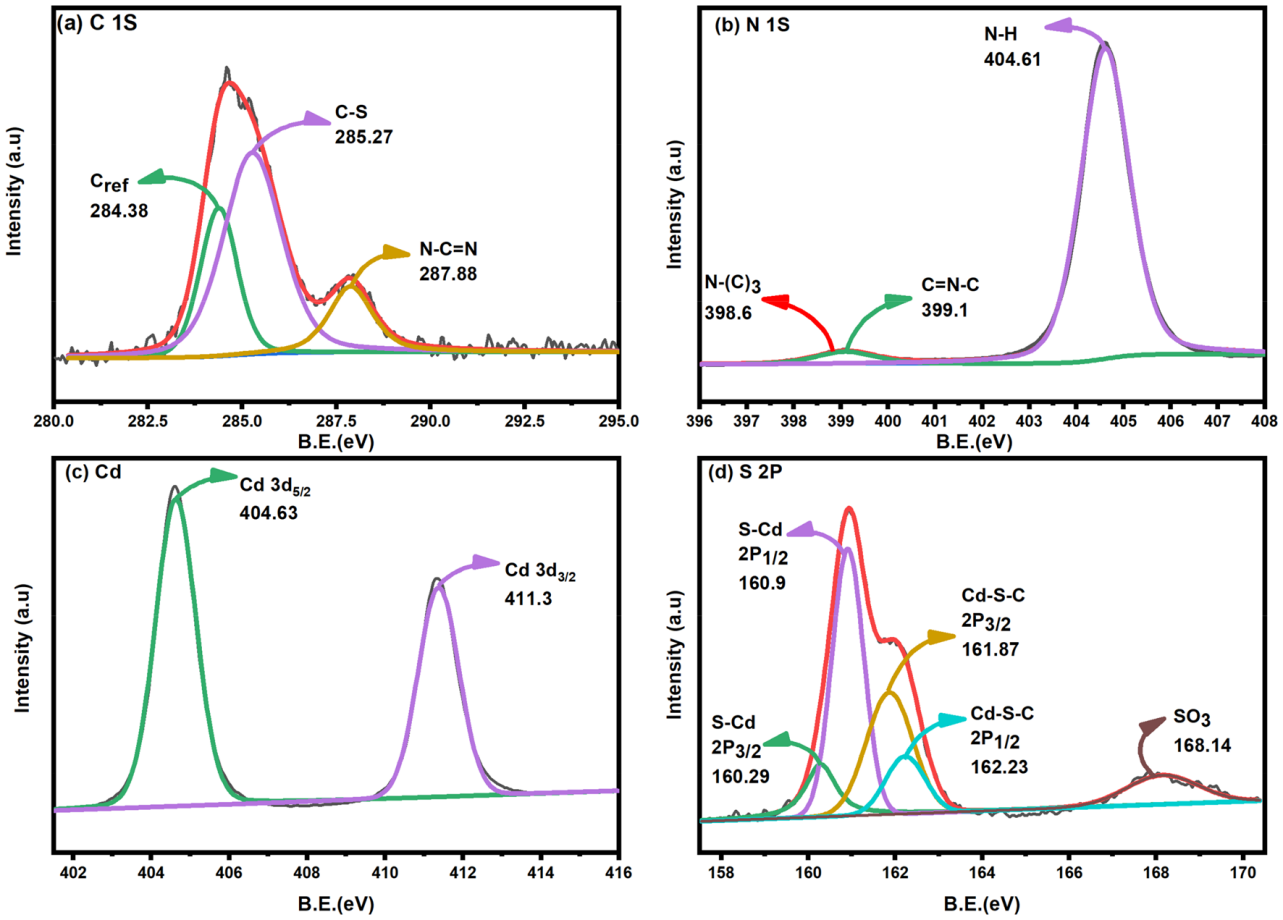
High deconvoluted XPS Spectra of S-Scheme heterojunction g-C_3_N_4_/CdS (**a**) C1*s*, (**b**) N 1*s*, (**c**) Cd 3*d*, (**d**) S 2*p*.

XPS was used to comprehensively understand the elemental makeup and chemical states of the S-Scheme-based g-C_3_N_4_/CdS with the acquisition parameters provided in supporting information in the Table S1. The C1*s* peak (Fig. 5a), which provides information about the carbon component of the material, was deconvoluted into three distinct peaks. The apparent two peaks at 284.38 eV and 287.88 eV were attributed to adventitious (Cref) and *sp*_2_ carbon linked to N-containing aromatic rings (C=N), correspondingly. The presence of *sp*_2_ C bonded to N-comprising aromatic rings is crucial as it has been well-established in the literature that this species contributes to the material's graphitic character and electrical conductivity. A third peak at 285.27 eV, like the C species found in the C–S bond, underscores the composite characteristics of the g-C_3_N_4_/CdS material. The C-S bond is essential in many composite materials, improving stability and electrical conductivity^45,46^. In addition to the C1s peak, the XPS analysis also revealed information about the nitrogen and sulfur components of the material. The N1s peak (as shown in Fig. 5b) was subjected to deconvolution, identifying three distinct peaks. The first peak, at an energy level of 398.6 eV, corresponds to tertiary Nitrogen units. The second peak, observed at 399.1 eV, is ascribed to *sp*2 hybridized aromatic Nitrogen atoms linked to carbon atoms in the graphite phase, specifically in the C=N–C configuration. The most substantial peak at 404.6 eV was identified as N–H bond^47^. The XPS characterization of the nitrogen component provides insight into the material's bonding environment and potential chemical reactivity. The Cd3*d* XPS spectrum of the g-C_3_N_4_/CdS composite revealed two intense peaks corresponding to Cd atoms in Cd-S bonds, with binding energies of 404.63 and 411.3 eV^48^,^49^. This information provides evidence of the presence of CdS in the material, which has important implications for its optical and electronic properties (Fig. 5c). The S2*p* spectrum was deconvoluted into two doublets, with the first doublet assigned to S species from CdS and the second doublet associated with S of C–S bonds. The small XPS (Fig. 5d) peak at 168.14 eV was found to correspond to S in sulfate groups^45^. This information provides a comprehensive understanding of the sulfur component in the material, which is critical for understanding its chemical and physical behavior.

**Figure 6:**
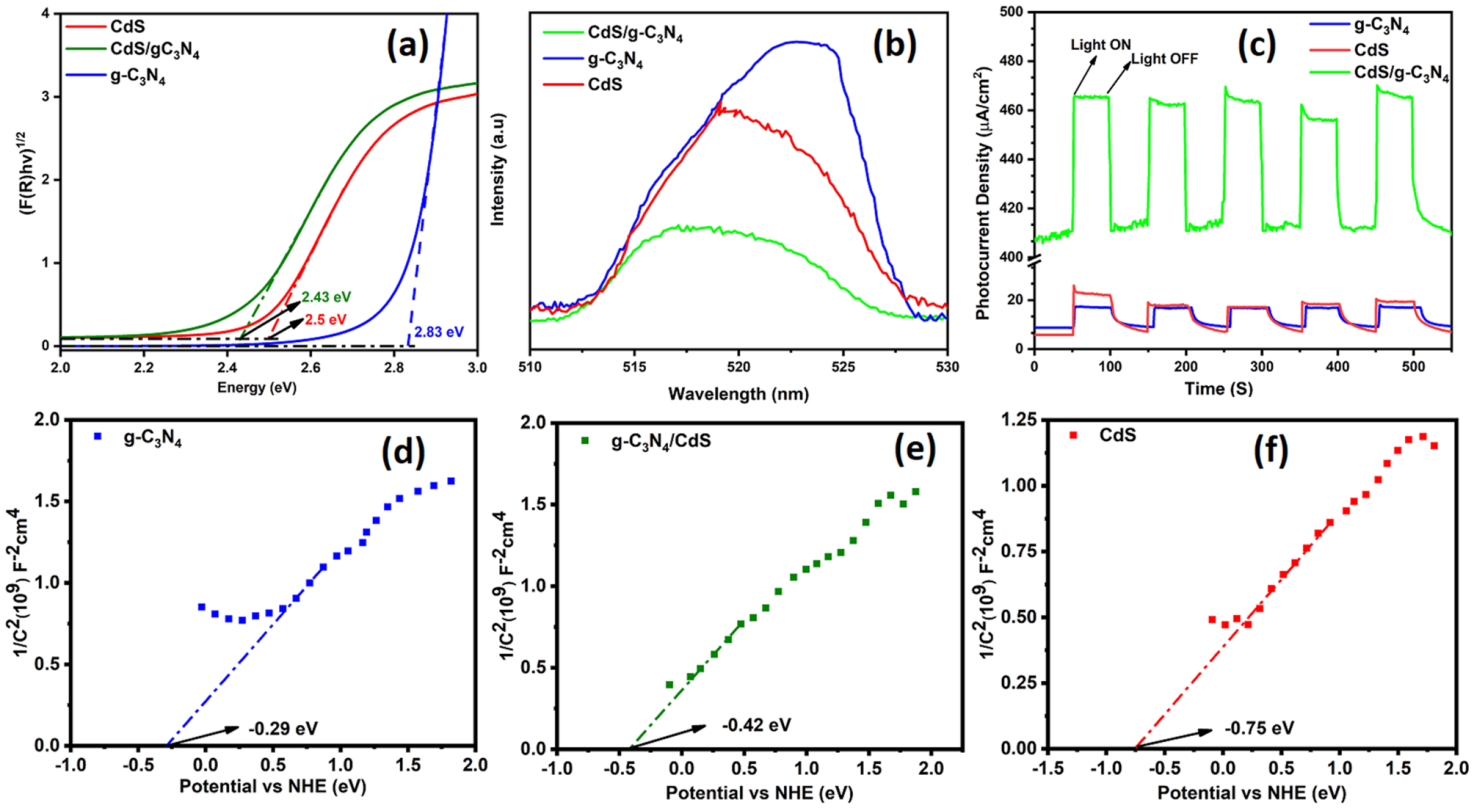
(**a**) DRS (**b**) PL (**c**) CA and (**d**–**f**) Mott–Schottky analysis of g-C_3_N_4_, CdS and g-C_3_N_4_/CdS.

The electronic properties of the material were determined using Kubelka–Munk transformation of DRS obtained data of the percentage of reflected light into absorbance and extrapolating the curve of Tauc plot (Fig. 6a). The obtained band gap values of 2.83 eV, 2.5 eV and 2.43 eV for g-C_3_N_4_, CdS, and g-C_3_N_4_/CdS S-Scheme composite respectively, confirms a decrease in band gap and the formation of heterojunction which implies an enhanced ability of the composite material to harness visible light, which is key for potential applications in photocatalysis or solar cells. Photoluminescence spectroscopy (PL) (Fig. 6b) further confirmed the electronic properties to study specifically the electrons and holes recombination rate of g-C_3_N_4_/CdS heterojunction. Using a 375 nm excitation wavelength, the results indicate the highest intensity peak for the g-C_3_N_4_, indicating the highest charges recombination rate, followed by CdS, which has a relatively lower intensity peak. The g-C_3_N_4_/CdS composite has the most minor intensity peak, indicating a decrease in charge recombination in the composite as compared to the pristine material. Many parameters, including as trap centers^50^, grain boundary defects^51^, band structure, and the mobility of carriers^52^, have been linked to this decreased emission intensity. Chronoamperometric chopping (CA) was employed to study the charges recombination and light response g-C_3_N_4_/CdS. The CA analysis (Fig. 6c) studied in a 0.5 M Na_2_SO_4_ gave insight into the photocurrent measurement of the g-C_3_N_4_/CdS heterostructure under chopped irradiation at supplied 0.7 V. The chopping rate was set at 50 s of light off–on rate. The results show that the composite material has a significantly enhanced photocurrent density of 55 uA/cm^2^, higher than its pristine materials, g-C_3_N_4_ and CdS, which had photocurrent densities of 8 uA/cm^2^ and 20 uA/cm^2^ respectively. The increased photocurrent density in the g-C_3_N_4_/CdS heterojunction system is indicative of a highly effective photogenerated charge transport and separation mechanism.

Additionally, the composite material showed an enhanced current density in the dark, further indicating improved charge transport and separation. The CA analysis supports the effectiveness of the g-C_3_N_4_/CdS heterojunction system in promoting efficient charge transport and separation, which is critical for applications in photovoltaic devices and photocatalysis. The improved results indicated by the transient photocurrent response supports the the S-Scheme electron pathway of g-C_3_N_4_/CdS, as supported by the reported work of Li et al.^53^. The Mott-Schottky analysis carried out at the applied frequency of 300 Hz and within potential window of − 0.5 V to + 0.5 V and the curves (Fig. 6d–f) were utilized to establish the relative locations of VB and CB of both the pure CdS and g-C_3_N_4_ materials, as well as the composite, and both of the materials were found to be n-type in this investigation. The predicted conduction band potentials of g-C_3_N_4_ and CdS were determined to be − 0.29 eV and − 0.75 eV against the NHE, respectively. The XPS VB analysis was done to determine the valance band positions of g-C_3_N_4_ and CdS and were determined to be 2.54 eV and 1.75 eV correspondingly to study the electron transport mechanism.

### Photocatalytic mineralisation

The investigation of photolytic degradation of 4-nitrophenol in natural sunlight was aimed to elucidate the stability of 4-nitrophenol upon exposure to solar radiation. The experiments were conducted in parallel at a sunlight intensity of 133.59 W/m^2^ determined by Metravi 207 Solar Power Meter. A 100-ppm aqueous solution of 4-nitrophenol was subjected to 120 min of solar irradiation, with ultraviolet–visible (UV–Vis) spectroscopy employed to analyze the solution at 30-min intervals. The findings (Fig. S2) demonstrated that 4-nitrophenol exhibits considerable stability under solar irradiation, with only minor conversion to its phenolate ion form observed.

**Figure 7:**
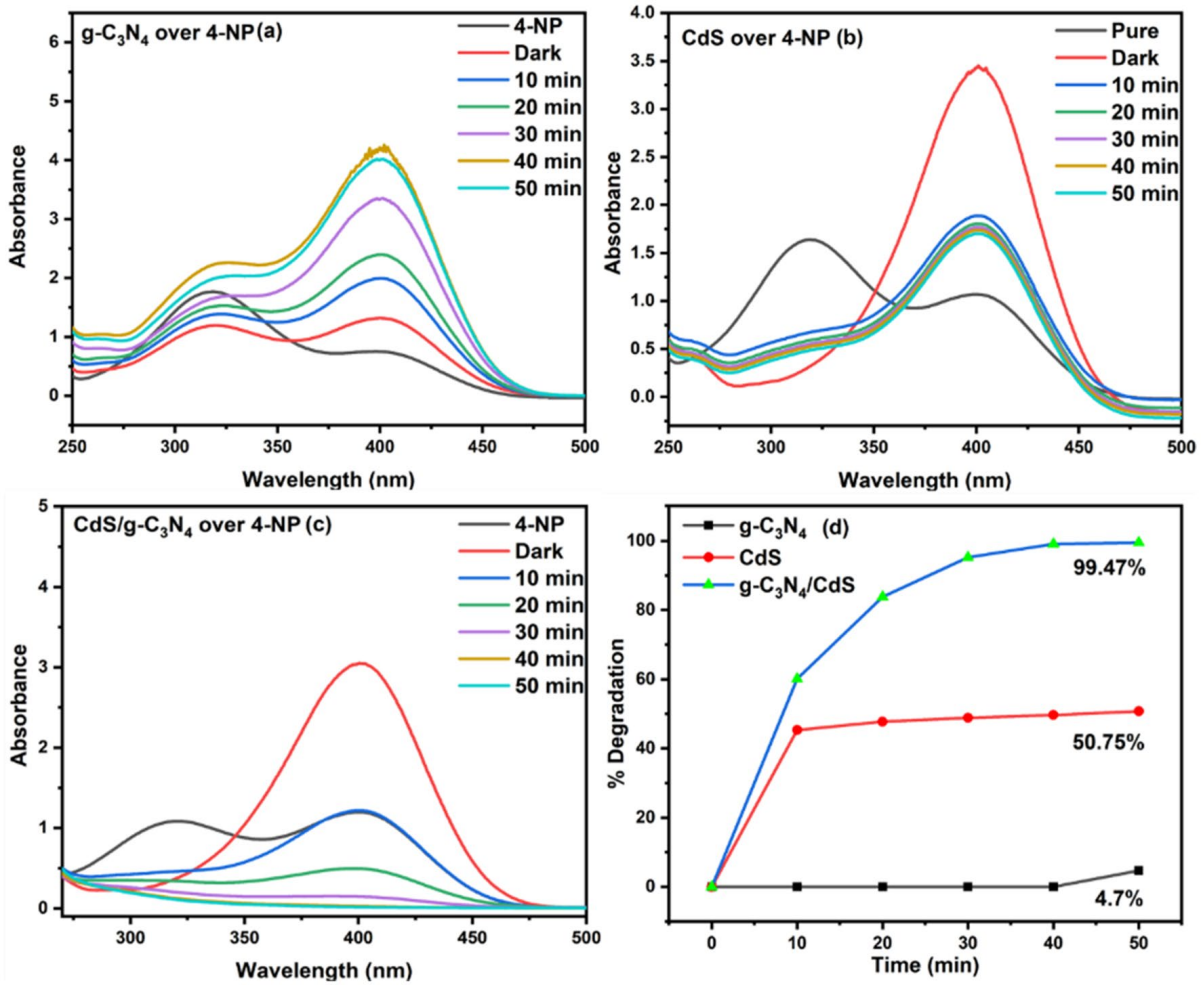
% Removal study of 4NP under (**a**) g-C3N4, (**b**) CdS and (**c**) g-C_3_N_4_/CdS.

The spectral data (Fig. 7) reveals the photocatalytic mineralization of 4NP. The graphs show the initial conversion of 4-NP to phenolate ion under dark conditions when it is allowed to form an adsorption–desorption equilibrium. From the graph (Fig. 7c), we can observe the decrease in intensity of 4-NP peak after 30 min of dark reaction. The maximum adsorption of phenol was calculated to be 67.4% at the equilibrium, followed by a continuous decrease in phenolate ion concentration when exposed to sunlight, indicative of 4-NP removal. For the g-C_3_N_4_ catalyst (Fig. 7a), 4-NP exhibited considerable stability, and even after 50 min, was predominantly converted to phenolate ion rather than being removed. The CdS catalyst (Fig. 7b) demonstrated a removal efficiency of 50.75% after a 50-min time interval. The most efficacious results (Fig. 7c) were obtained using the composite photocatalyst g-C_3_N_4_/CdS, which exhibited a removal efficiency of 99.47% after a 50-min exposure period. The same procedure was applied for the study of 2NP as well. It gave the 2NP removal of 0%, 30%, and 84% using g-C_3_N_4_, CdS and g-C_3_N_4_/CdS respectively (Fig. S3).

This percentage removal (Fig. 7 and Fig. S3) do not conclusively verify the complete elimination of 4-NP and 2NP from the solution, respectively. To determine if it had been changed into another substance or experienced complete or partial mineralization i.e., degradation of Nitrophenols to CO_2_ and H_2_O, a Total Organic Carbon (TOC) analysis (Fig. S4) was performed. Measurements were obtained at time intervals of 20, 40, and 50 min with the optimized composite catalyst g-C_3_N_4_/CdS, yielding corresponding carbon removal percentages of 19.99%, 32.84%, and 78.61% for 4NP and 6.94%, 27.74%, and 56.51% for 2NP respectively.

Using a 4-nitrophenol (4-NP) solution, we studied how changing the percentage of g-C_3_N_4_ in the g-C_3_N_4_/CdS affected the photodegradation efficiency. The results (Fig. S5a) revealed that the composite confining 30% g-C_3_N_4_ exhibited the lowest efficiency (73.11%), followed by the composites with 20% and 10% g-C_3_N_4_. The highest efficiency was achieved with the 15% g-C_3_N_4_/CdS composite. These findings suggest that cadmium sulfide (CdS) is the primary light-responsive component in the composite. Beyond the optimum concentration, the secondary catalyst (g-C_3_N_4_) appears to overshadow CdS, resulting in reduced light absorption by CdS and, consequently, diminished photocatalytic activity. It can be concluded that the optimal performance is attained using a 15% g-C_3_N_4_/CdS heterojunction catalyst. The catalyst's recyclability was examined (Figs. S5b and S5c), showing substantial stability that attests to the successful creation of a heterojunction that supported the catalyst's stability and, as a result, enabled effective charge separation. The catalyst's stability was evaluated for up to four cycles for 4-NP and 2-NP. The catalyst g-C_3_N_4_/CdS retained its morphology even after four cycles as shown in SEM images in the supplementary information (Fig. S6) and structural stability as indicated by the retainment of peaks in XRD (supporting information Fig. S7).

### Mechanism of enhanced photocatalytic mineralisation

There are two commonly accepted methods to explain the movement of charge carriers over the interface between two semiconductors: the heterojunction type II and the step-scheme (S-scheme) mechanism. If we suppose there is a heterojunction type II in the current system, e^−^ and h^+^ will be separated across the interface of CdS and g-C_3_N_4_. The photogenerated e^−^ will be conserved on CB of g-C_3_N_4_ and h^+^ on VB of CdS but it will have several drawbacks: (1) electrostatic repulsion and attraction will hinder charge transfer and promote recombination; (2) the active electrons and holes will be located on lower energy bands i.e., − 0.29 eV (Fig. 6d) and + 1.75 eV (supporting information S8b) in the current system, limiting their redox ability making it impossible to very low production of superoxide radicals (O_2_·¯) and hydroxyl radicals (·OH), which occurs at − 0.33 eV and + 2.32 eV respectively; (3) the holes on CdS cause photo corrosion, and water oxidation cannot occur. While in S-scheme, electrons and holes are transferred within the same component, avoiding these problems and enhancing photocatalytic performance, as supported by the work of Li et al.^54^ and Cai et al.^55^.

**Figure 8:**
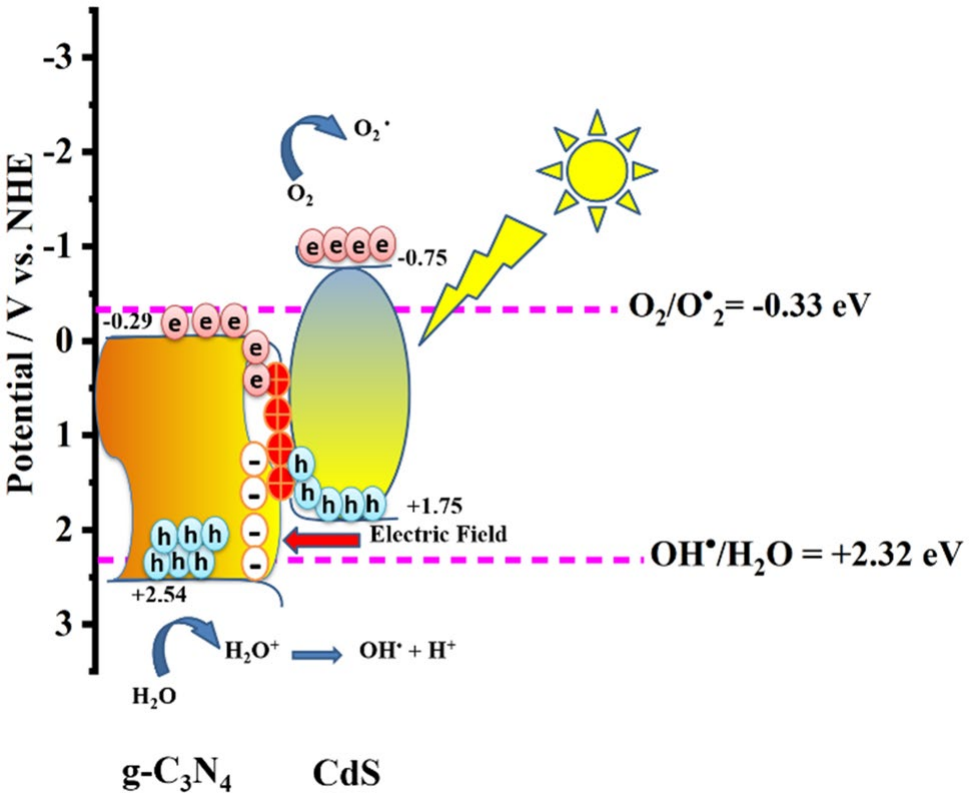
S-scheme Mechanism of g-C_3_N_4_/CdS.

Developing a heterojunction helps faster electron mobility at the heterointerface from the low work function catalyst, CdS, to the high work function catalyst, g-C_3_N_4_. This electron transfer creates an e^−^-rich zone on the g-C_3_N_4_ side and a positively charged layer on the CdS side, generating an electric field (Fig. 8). The process of hole transport occurring on the valence band (VB) of CdS and electron transport occurring on the CB of g-C_3_N_4_ is facilitated by the polarization of the electrical double layer and the accumulation of charge carriers at the edge. This accumulation subsequently leads to bending the VB maximum and CB minimum. This particular methodology facilitates the preservation of vacancies on the valence band of g-C_3_N_4_ and electrons on the CB of CdS, hence enabling efficient segregation of photoinduced electrons and holes. Photocatalytic processes, such as the reduction of O_2_ to ^·^O_2_ and the oxidation of H_2_O to ^·^OH, rely on the division and distribution of photoinduced electrons and holes, that are aided by the built-in electric field. This charge flow mechanism is corroborated by the literature, as the minimum potential required for the production of superoxide radicals (O_2_·¯) and hydroxyl radicals (·OH), which is − 0.33 eV and + 2.32 eV respectively^55,56^ and cannot be achieved by g-C_3_N_4_ or CdS alone, given their respective conduction band position at − 0.29 eV (Fig. 6d) and valence band position at + 1.75 eV as determined by XPS VB (supporting information fig. S8). Additionally, releasing oxidative h^+^ on the VB of CdS during recombination mitigate the oxidation of S^2−^ ions in the geometry, stabilizing the material. If it was a type II mechanism, the accretion of holes at the VB of CdS would have caused the photo-corrosion of the material via oxidation of S^2−^, but experimentally, we can see an enhanced stability and efficiency of the synthesised product, supporting our claim of the S-Scheme electron transfer pathway. This explanation of the heterojunction operating via an S-scheme provides a plausible account of the g-C_3_N_4_/CdS interface’s function as a photocatalyst.

## Conclusion

In summary, this study developed and characterized S-scheme g-C_3_N_4_/CdS composites system, and to the best of our knowledge; we reported its first-time usage in efficient photocatalytic degradation of nitrophenol pollutants. The composite demonstrated improved charge separation with the help of built-in electric field preventing the photo-corrosion of CdS, higher visible light absorption, and good structural solidity; as supported by enhanced light absorption and current response of 55 uA/cm^2^ by CA, fine nano-heterojunction formation and charge carrier’s pathway confirmation by TEM and mott-schotky, and sufficient separation of e^−^ and h^+^ by PL studies. The high energy e^−^ (− 0.75 eV) produced at the CB of CdS and h^+^ (+ 2.54 eV) led to the production of O_2_·¯ and ·OH. The lower band gap of 2.43 eV as calculated by DRS confirmed the visible light absorption by g-C_3_N_4_/CdS. The 2D flexible morphology of CdS coupled with g-C_3_N_4_ aided the charge transport and enhanced stability of CdS. Under natural sunlight, the optimized 15% g-C_3_N_4_/CdS catalyst achieved degradation efficiencies of 99.4% for 4-nitrophenol and 84% for 2-nitrophenol within 50 min due. TOC analysis further confirmed mineralization efficiencies up to 79%. The catalyst also exhibited recyclability of an average 99% degradation over four cycles with its structural stability confirmed by XRD and SEM after the experiment. It was made possible for an efficient photoinduced charge transfer mechanism—S-Scheme between g-C_3_N_4_ and CdS by the development of a well-defined heterojunction contact. All things considered; the S-scheme heterojunction design presents a successful method for creating highly effective photocatalysts with enhanced stability against photocorrosion to have potential uses in environmental cleanup. Further work could explore large-scale synthesis methods and wastewater treatment under natural conditions.

### Supplementary Information

Supplementary Information.

## Data Availability

The datasets generated during and/or analyzed during the current study are available from the corresponding author upon reasonable request.
