# Supplementary material for: Development of stable S-scheme 2D–2D g-C3N4/CdS nanoheterojunction arrays for enhanced visible light photomineralisation of nitrophenol priority water pollutants
Source: Sci Rep. 2024 Feb 5;14:2897. doi: 10.1038/s41598-024-52950-3 (PMC10844285; doi:10.1038/s41598-024-52950-3)
Supplement: Supplementary file 1 — Supplementary Information. [file 41598_2024_52950_MOESM1_ESM.docx]

**Supporting Information**

**Development of Stable S-Scheme 2D-2D g-C_3_N_4_/CdS** **Nanoheterojunction Arrays for Enhanced Visible Light Photomineralisation of Nitrophenol Priority Water Pollutants**

Muhammad Saad^1,2,3^, Ali Bahadur^4,5^*, Shahid Iqbal^6^*, Sajid Mahmood^6,7^, Muhammad Tayyab^8^, Matar Alshalwi^9^, Mazloom Shah^10^

*^1^Centre for Organic and Nanohybrid Electronics, Silesian University of Technology, Konarskiego 22B, 44-100 Gliwice, Poland.*

*^2^Joint Doctoral School, Silesian University of Technology, Akademicka 2A, 44-100 Gliwice, Poland.*

*^3^Department of Chemistry, School of Natural Sciences (SNS), National University of Science and Technology (NUST), H-12, Islamabad, 46000, Pakistan.*

*^4^Department of Chemistry, College of Science, Mathematics, and Technology, Wenzhou-Kean University, Wenzhou 325060, Zhejiang Province, China.*

*^5^Dorothy and George Hennings College of Science, Mathematics and Technology, Kean University, 1000 Morris Ave, Union, NJ 07083, USA.*

*^6^Nottingham Ningbo China Beacons of Excellence Research and Innovation Institute, University of Nottingham Ningbo China, Ningbo 315100, China.*

*^7^Functional Materials Group, Gulf University for Science and Technology, Mishref 32093, Kuwait.*

*^8^Department of Chemical and Life Sciences, Qurtuba University of Science and Information Technology, Dera Ismail Khan, Pakistan.*

*^9^Department of Chemistry, Collage of Science, King Saud University, PO Box 2455, Riyadh 11541, Saudi Arabia.*

*^10^Department of Chemistry, Faculty of Science, Grand Asian University Sialkot, Pakistan.*

***To whom corresponding should be addressed**

[shahidiqbal@hzu.edu.cn](mailto:shahidiqbal@hzu.edu.cn) (Shahid Iqbal) and [abahadur@wku.edu.cn](mailto:abahadur@wku.edu.cn) (Ali Bahadur)

**S1. Reagents Used:**

All the reagents and raw materials used in the current study were of analytical quality and were used without any additional purification from their original form. Cadmium acetate dihydrate, supplied by Unichem, and thiourea, procured from Sigma Aldrich, functioned as the sources of Cd and S, respectively, in the reaction for CdS synthesis. Ethylenediamine, acquired from Daejung Chemicals, was employed as both solvent and complexing agent. Furthermore, melamine, sourced from Sigma Aldrich, and urea, likewise obtained from Sigma Aldrich, served as precursor reagents for graphitic carbon nitride (g-C3N4) synthesis.


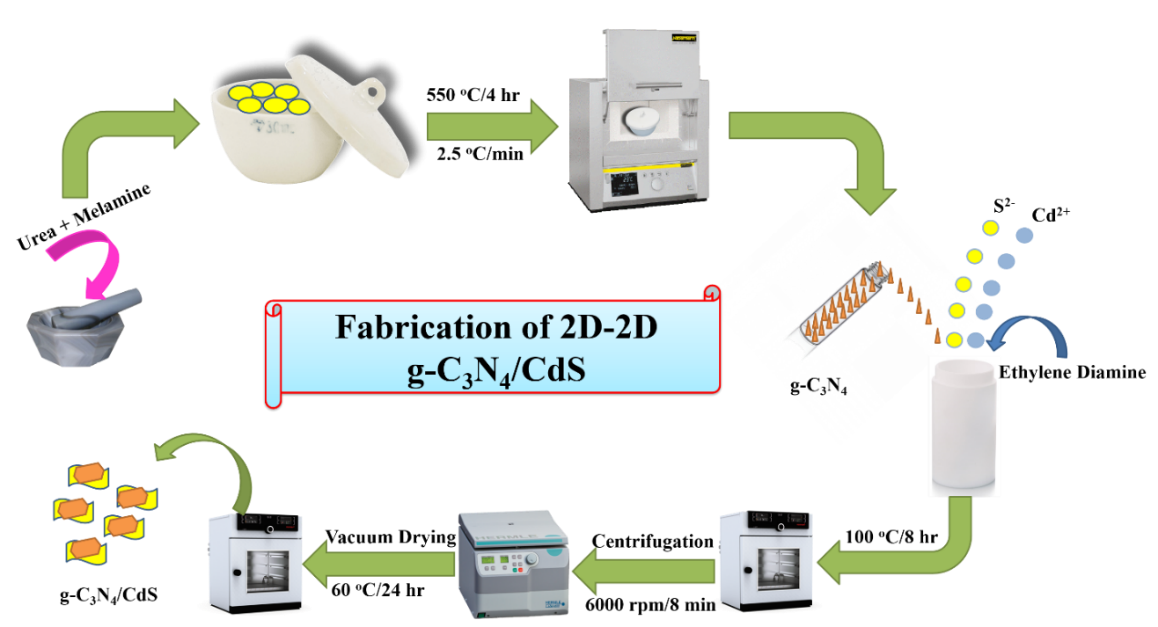


**Figure S1.** Synthesis and Fabrication of 2D-2D g-C3N4/CdS Nanoheterojunction.

**Table S1** XPS Acquisition Parameters of gC3N4/CdS

| **Parameter** | **Details** |
| --- | --- |
| Total acquisition time | 1 mins 0.2 secs |
| Number of Scans | 3 |
| Source Gun Type | Al K Alpha |
| Spot Size | 400 µm |
| Lens Mode | Standard |
| Analyzer Mode | CAE : Pass Energy 30.0 eV |
| Energy Step Size | 0.050 eV |
| Number of Energy Steps | 401 |


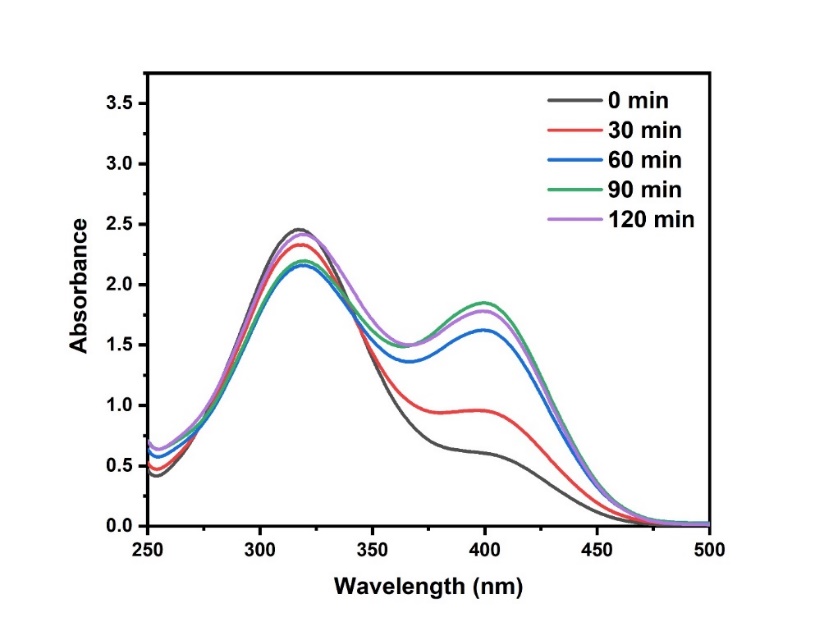


**Figure S2** Photolysis Study of 4-Nitrophenol.


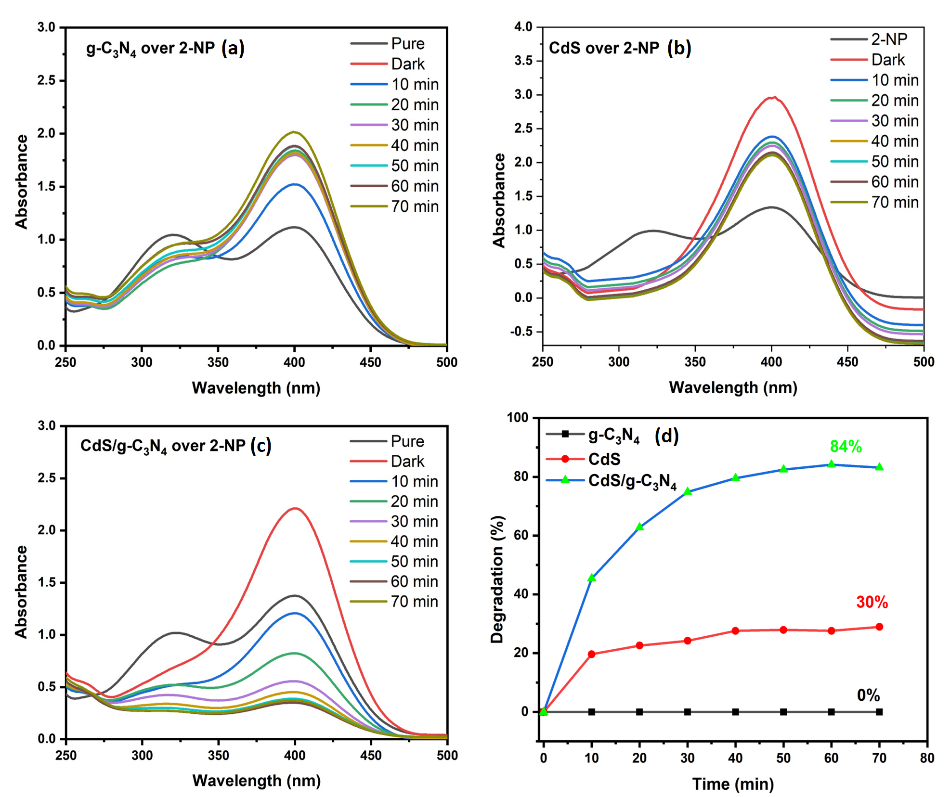


**Figure S3** % Removal of 2NP using (a)g-C3N4, (b)CdS and (c)g-C3N4/CdS (d)Comparison.


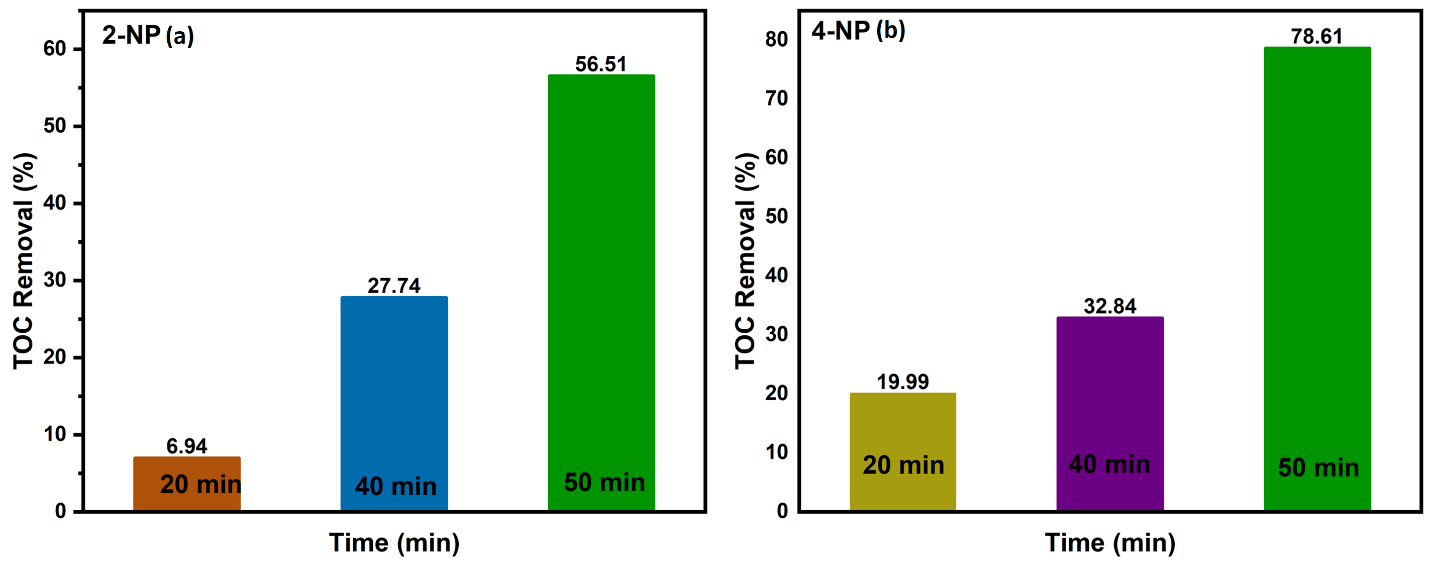


**Figure S4** TOC Removal Study of (a)2NP and (b)4NP under g-C_3_N_4_/CdS.


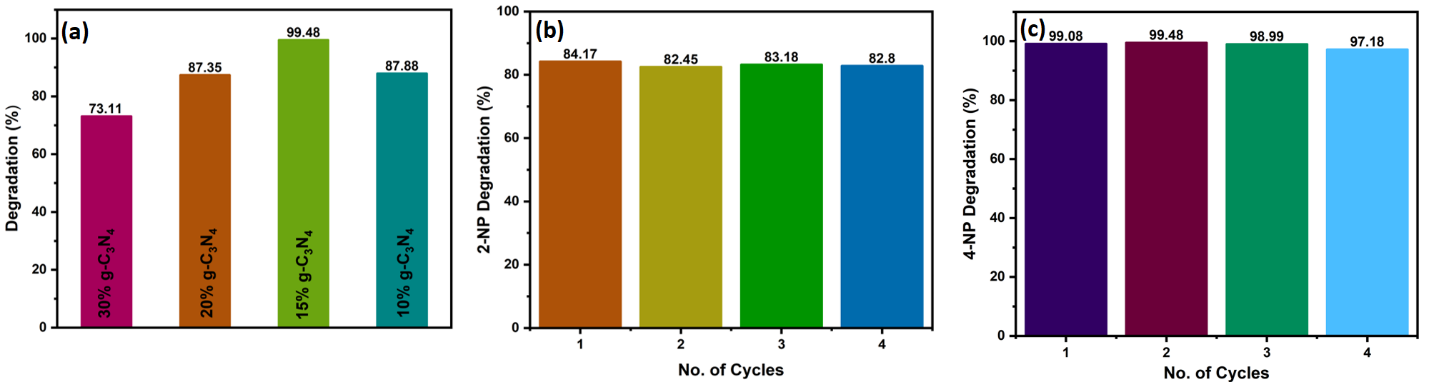


**Figure S5** (a) Catalyst's Compositional Analysis over 4-Nitrophenol, (b) Reusability study on 2NP, and (c) Reusability study on 4NP.





**Figure S6.** SEM image of g-C3N4/CdS after 4 cycles





**Figure S7.** XRD comparison of g-C_3_N_4_/CdS recyclability studies after four cycles


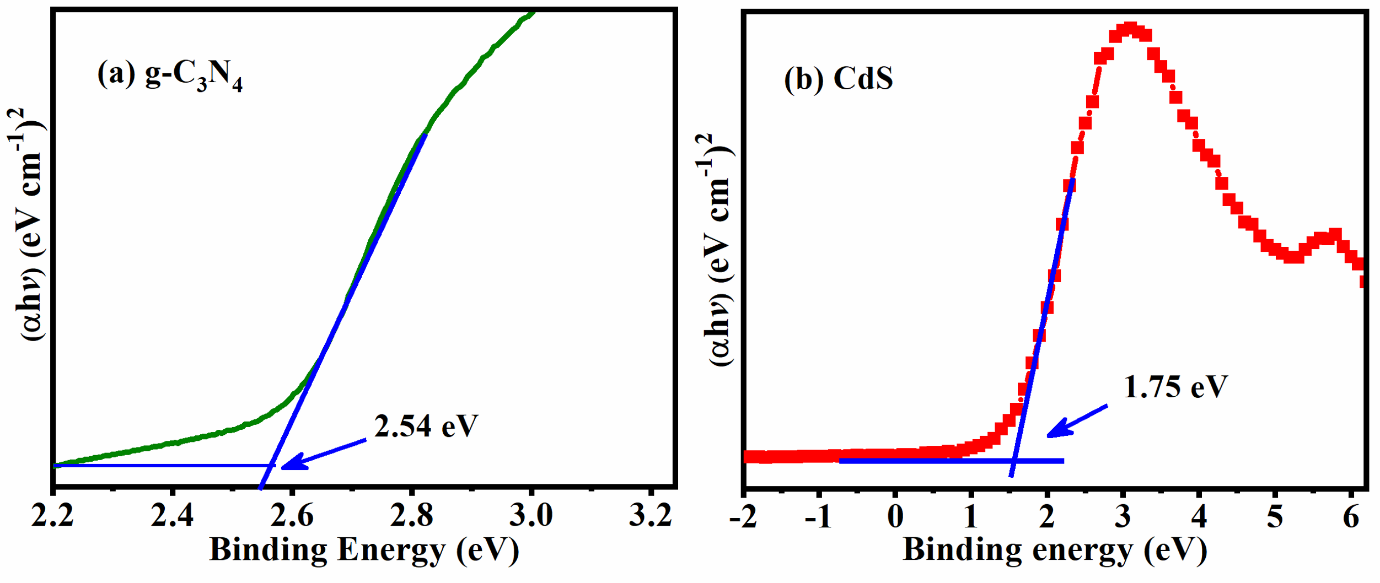


**Figure S8.** XPS VB (a)g-C_3_N_4_ and (b)CdS
